# Elevational Ranges of Montane Birds and Deforestation in the Western Andes of Colombia

**DOI:** 10.1371/journal.pone.0143311

**Published:** 2015-12-07

**Authors:** Natalia Ocampo-Peñuela, Stuart L. Pimm

**Affiliations:** Nicholas School of the Environment, Duke University, Durham, North Carolina, United States of America; University of Sydney, AUSTRALIA

## Abstract

Deforestation causes habitat loss, fragmentation, degradation, and can ultimately cause extinction of the remnant species. Tropical montane birds face these threats with the added natural vulnerability of narrower elevational ranges and higher specialization than lowland species. Recent studies assess the impact of present and future global climate change on species’ ranges, but only a few of these evaluate the potentially confounding effect of lowland deforestation on species elevational distributions. In the Western Andes of Colombia, an important biodiversity hotspot, we evaluated the effects of deforestation on the elevational ranges of montane birds along altitudinal transects. Using point counts and mist-nets, we surveyed six altitudinal transects spanning 2200 to 2800m. Three transects were forested from 2200 to 2800m, and three were partially deforested with forest cover only above 2400m. We compared abundance-weighted mean elevation, minimum elevation, and elevational range width. In addition to analysing the effect of deforestation on 134 species, we tested its impact within trophic guilds and habitat preference groups. Abundance-weighted mean and minimum elevations were not significantly different between forested and partially deforested transects. Range width was marginally different: as expected, ranges were larger in forested transects. Species in different trophic guilds and habitat preference categories showed different trends. These results suggest that deforestation may affect species’ elevational ranges, even within the forest that remains. Climate change will likely exacerbate harmful impacts of deforestation on species’ elevational distributions. Future conservation strategies need to account for this by protecting connected forest tracts across a wide range of elevations.

## Introduction

Deforestation causes habitat loss, fragmentation, and degradation, and can ultimately cause the extinction of the remnant species [1–5]. In the tropics, these are the main drivers of immediate and delayed extinction in birds [6]. Researchers have studied the effects of habitat loss and fragmentation and identified edge effects [7–9], loss of landscape connectivity [10], reductions of population sizes [11], and ultimately influence on genetic structures of populations [12, 13] as the major contributors to species vulnerability to extinction. Endangerment and extinction are likely not the result of one of these causes, but rather a synergy of processes which will likely be exacerbated by climate change [14].

Geographic ranges of species have been used to study species vulnerability [15] and inform conservation decisions with a wide range of success [16–18]. From previous studies, we know that species with the smallest geographical ranges, such as those found on mountains or islands [16], are the most threatened with extinction [2, 19], and will be more severely affected by climate change [20, 21]. Remnant populations of birds, for instance, are often restricted to mountain ranges [22] and thus we expect the larger number of future extinctions to be on mountain tops [23, 24]. Projections of range contraction in response to climate change anticipate 400 bird species reducing their ranges by half, although in the tropics the main cause of range contractions remains land-use change [25].

We know that specialized bird species disappear soon after the disturbance from deforested areas, and slowly from remaining forest fragments [3]. Here, we explore one neglected question: do species adjust their elevational ranges in response to the loss of natural habitat below them? We compare species ranges from completely forested transects to those that have lost forest from lower elevations. We anticipated differences, expecting that across comparable elevational spans species would have larger ranges when there was forest below than when there was not. Some species might well be absent near the lower end of their range if there is a forest edge because of the added harm that befalls them there [26, 27].

Our motivation is both to add to the body of literature on the effects of habitat loss and to understand the effect of lowland deforestation on the elevational ranges of species. An increasing number of studies search for long-term changes in species distributions along elevational transects driven by changes in the global climate [20, 28–30]. We still need to understand the potentially confounding effect of lowland deforestation in the face of climate change [14].

We chose the Northern Andes as our study area as it is one of the most diverse areas in the world [31, 32]. In the Western Andes of Colombia, concentrations of endemic and threatened birds peak at 46 species in specific areas [17] and the diversity of other taxa is also high [33].

In addition, Neotropical birds, and especially those preferring higher elevations, have narrower ranges than birds in other regions, making them more vulnerable to extinction [21, 24, 34–36]. When faced with habitat loss and climate change, species with narrow niches and poor dispersal ability will be severely affected [37], while generalists and mobile species may thrive [38].

We evaluate the effects of deforestation on the elevational ranges of montane birds, specifically their mean and minimum elevations, and elevational range width. In addition to analysing the effect on species, we tested its impact across different trophic guilds and habitat preferences. We are unaware of previous studies designed to use extensive elevational transect fieldwork to evaluate impacts of lowland deforestation in the species that remain at the still forested higher elevations.

Previous studies have found bird extinction risk increases as elevational ranges become smaller. We predicted that deforestation in the lower portion of an altitudinal transect might disproportionately impact the elevational ranges of bird species with narrow elevational ranges and those that prefer forest interiors, or depend on seasonal food resources (such as nectar or fruit). Species with narrower elevational ranges lack the option to find refuges from anthropogenic disturbances, increasing their extinction risk [21, 34]. We expected species’ mean and minimum elevations to be at higher elevations in transects lacking forest in the lowlands, since some montane birds are altitudinal migrants and will likely be forced to move up in search of resources, especially fruit and nectar-eating birds. As for elevational range width, we expected a contraction in partially deforested transects, in accordance to previous studies that predict range contractions due to habitat loss [39].

## Materials and Methods

### Study area

Colombia’s Andes have three main cordilleras; the Western lies between the Pacific Ocean and the Cauca Valley. The Western Andes of Colombia have the highest diversity and endemism of birds [17, 40, 41], rodents, bats, butterflies, and frogs in the country [33, 40]. The east and west slopes of this cordillera differ in their climate and conservation status. The east slope is located in a rain shadow, making its climate more arid than the moist west slope which receives winds from the Pacific Ocean [33]. Most of the human population in the Western Andes lives on its east slope and causes severe disturbances from agricultural use and cattle grazing [40]. Forests remain large and connected along the west slope, due to the lack of road access [42, 43], and the presence of groups of political insurgents.

**Fig 1:**
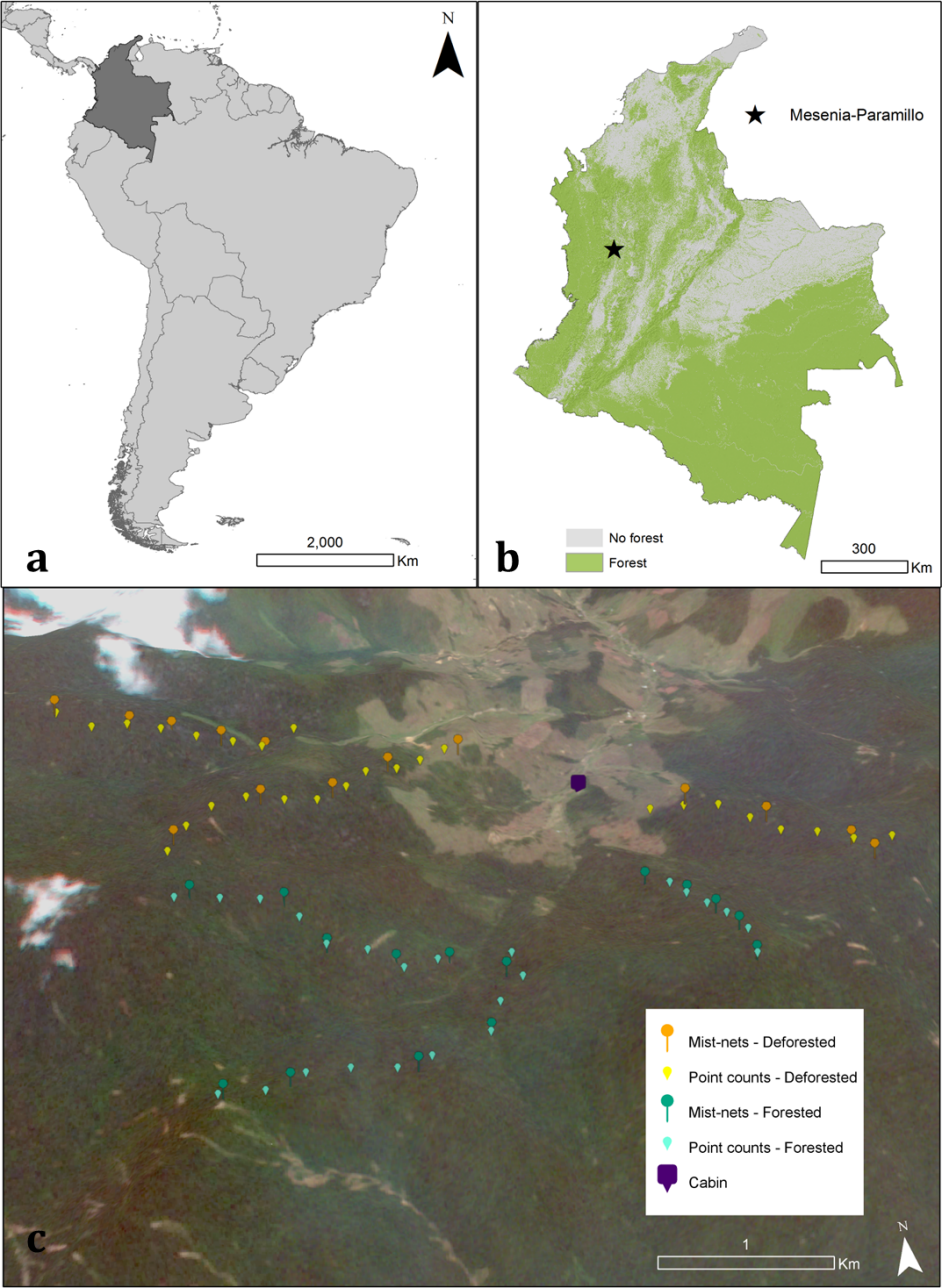
Study area. a) Location of Colombia in South America. b) Location of Mesenia-Paramillo Nature Reserve over a forest cover map from Hansen, Potapov [45]. c) Location of forested and partially deforested transects and their respective mist-net and point count locations in Mesenia-Paramillo Reserve. Background image from satellite RapidEye for dates 28/12/2013-01/04/2014. Displayed here with permission from Saving Species (www.savingspecies.org) and in line with agreement from seller BlackBridge.

Our study took place in the Mesenia-Paramillo Nature Reserve (henceforth Mesenia-Paramillo) located in the municipality of Jardín, in Antioquia (Fig 1A and 1B). It lies between Tatamá and Orquídeas National Parks, and currently covers 3000 ha. Mesenia-Paramillo was purchased by Fundación Colibrí in 2007 and continues to expand its conservation area. Elevations span 2200 to 3200 meters above sea level (masl), ranging from Andean cloud forests to paramos. The reserve conserves large concentrations of endemic and small-ranged bird species [17], as well as significant diversity of other taxa [44].

### Field methods

Between April 2014 and March 2015, we surveyed six altitudinal transects for bird species richness and abundance using point counts and mist-nets. Three transects had forest from 2200 to 2800 m (Fig 1C, teal). The other three transects only had forest between 2400 and 2800 m; between 2200 and 2400 there were pastures (Fig 1C, orange). Henceforth, we will refer to these transects as “forested” and “partially deforested”, respectively. Partially deforested transects have not had forest cover for at least 40 years, with no forest regeneration, as we observed in historical satellite imagery. In a similar way, forested transects have remained forested for at least 40 years. The forest in all transects had no obvious differences in plant structure and composition, but we acknowledge the fact that other factors could be influencing bird communities in these transects. We conducted all surveys inside forest, excluding paramos and pastures. Although we collected bird data from 2200 to 2800m, we only compared bird data from the forested part of both transects (2400-2800m) because we were interested in the changes to the elevational ranges of birds in the remaining forest. Bird diversity and structure was strikingly different below 2400m due to the lack of forest in partially deforested transects.

Point counts are generally the preferred method for assessing bird richness and abundance [46]. Although measuring distance to each observation is recommended [47], this was not possible in the dense and complex cloud forest of the study site, especially because montane birds often move in mixed species flocks [48]. To keep abundance estimates as accurate as possible, we had fixed-distance point counts with a 25 m radius.

We placed point counts every 150 linear meters spanning 2400 to 2800 m following methods suggested by [49]. We measured the elevations and distance using a handheld GPS. Due to differences in topography, some transects had more point counts than others: the three forested transects had a total of 24 point counts while partially deforested transects had a combined 27 point counts. Point survey started at 0600 h and generally went until 1000 h. In each point count, a single observer recorded every bird seen or heard during 12 minutes; within and outside the fixed distance of 25 m. Observers were experienced with the region’s birds and especially their calls. We surveyed each point 20 times, alternating the order of the surveys to keep the effort standard. To control for unknown impacts of altitudinal migrations, repetitions of point count surveys for all six transects were distributed along one year as follows: 8 repetitions between May and October of 2014, 6 in November and December of 2014, and an additional 6 between January and February of 2015. We did not survey during rain or severe winds.

At each transect we had five mist-netting stations located every 100 altitudinal meters (2400, 2500, 2600, 2700, and 2800) inside forest. We operated fifteen 12 m, 36mm mesh size, mist-nets at each station for a total of 600 net/hours, generally lasting 4 to 5 days. Operating protocol generally followed [49]. We mist-netted on 2 transects at the same time, using two separate field teams, between April and September of 2014. We opened nets before sunrise at 0530 and closed them before sunset at 1730, except in the presence of rain or severe winds. One experienced bander and an assistant checked nets every 30 minutes and identified, banded, and released each captured individual.

### Data preparation

We used two different types of data to answer our research questions: species richness, and species relative abundance. For species richness (presence/absence), we used data from both point counts (inside and outside the fixed distance) and mist-nets. When analysing species relative abundance, we only employed data from inside the 25m point count radius because this method had more repetitions and thus better seasonal representation. Additionally, we only captured 18 species in mist-nets that were not observed in point counts. Most of these mist-net exclusive species we captured only once.

For all analyses, we grouped the three forested transects, and the three partially deforested transects and treated them as forested and partially deforested respectively. For each point count, we considered relative abundance as the sum of the counts on the 20 repetitions. We compared the two types of transects based on elevational range variables: mean, minimum, and elevational range width. For the mean elevation, we used abundance from point counts to weight the mean by taking the sum of each count, multiplying it by its elevation, and then dividing that sum by the sum of all elevations. Abundance-weighted mean elevations are more appropriate for understanding the distribution of the species along the altitudinal transect [23]. The minimum and maximum elevations were the extreme observations of a species on any transect replicate. The elevational range width is the subtraction of the minimum elevation from the maximum. We determined range width for each species as the average of the three transect replicates.

Following analyses at the species level, we compared ecologically meaningful groups classifying each species in a specific trophic guild and habitat preference category. We assigned groups based on information found in the “Handbook of the Birds of the World Alive” [50], and the “Guide to the Birds of Colombia” [51]. We assigned a trophic guild category to each species based on accounts of its major food source: nectar, fruit, seeds, insects, or meat. For analyses, we excluded raptors that have very broad ranges and are likely not as constrained by changes in land cover at different altitudes. We also excluded seed eating species because this category had only 11 species.

We also classified species into one of four habitat categories according to their habitat use accounts from [50]: interior (only when the word “interior” was explicitly used and when the text indicated edge avoidance), edge (only when the word “edge” was explicitly used), forest (when the species uses forest but there is no statement of interior or edge), and non-forest (when explicitly stated as not using forest). We excluded the non-forest category because it only had two species, both present only in partially deforested transects.

### Data analysis

Using 134 species found in both forested and partially deforested transects, we tested if species’ elevational ranges were higher in partially deforested transects. We ran one-tailed, one-factor ANOVAs for variables: abundance-weighted mean, minimum elevation, and range width using the statistics package R [52]. For the range width, we excluded species that we only recorded once. This process eliminated 37 species from forested transects, and 31 from partially deforested transects.

We also tested if the presence of forest below 2400 m affected species in the various trophic guilds and habitat preference categories differently. We analysed these effects using two-factor ANOVAs in R [52]. Using the previously established elevation variables (abundance-weighted mean, minimum, and elevational range width) as response variables, we evaluated the factors trophic guild/habitat preference, and presence of forest below 2400 m. When the ANOVAs were at least marginally significant (p<0.1), we ran TukeyHSD tests for more detail on the effect analysed. We treated range width as in the species analyses and eliminated zero values.

## Results

During our field season, including point counts and mist-nets, we recorded 227 species in 39 families in Mesenia-Paramillo (S1 Table, S6 Table). Nine of the species recorded are deemed threatened by the International Union for the Conservation of Nature [15]: the critically endangered Munchique Wood-wren (*Henicorhina negreti*), the endangered Chestnut-bellied Flowerpiercer (*Diglossa gloriosissima*), Yellow-eared Parrot (*Ognorhynchus icterotis*), Magdalena Tapaculo (*Scytalopus rodriguezi*), and the vulnerable Bicoloured Antvireo (*Dysithamnus occidentalis*), Tanager Finch (*Oreothraupis arremonops*), Ruddy Pigeon (*Patagioenas subvinacea*), White-capped Tanager (*Sericossypha albocristata*), and Red-bellied Grackle (*Hypopyrrhus pyrohypogaster*). Of these threatened species, four are endemic to Colombia (Munchique Wood-wren, Chestnut-bellied Flowerpiercer, Magdalena Tapaculo, Red-bellied Grackle), and an additional three non-threatened species are also endemic: Stiles’ Tapaculo (*Scytalopus stilesi*), Paramo Tapaculo (*Scytalopus canus*), and Chestnut Wood-quail (*Odontophorus hyperythrus*). As far as we know, this is the first time the Magdalena Tapaculo has been recorded this far north.

Forested transects had 179 species (171 in point counts and mist-nets, 8 incidental observations outside standardized point counts) in 37 families, while partially deforested transects had 192 species (177 in point counts and mist-nets, 15 incidental observations outside standardized point counts) in 36 families. Differences between species composition and families of the two types of transects were not statistically significant (two sample t-tests, t = 0.409, df = 37, p-value = 0.685). Of the endemic species, five were present in both types of transects (Chestnut Wood-quail, Paramo Tapaculo, Munchique Wood-wren, Chestnut-bellied Flowerpiercer, Red-bellied Grackle), and two were unique to forested transects (Magdalena Tapaculo, Stiles’ Tapaculo).

**Fig 2:**
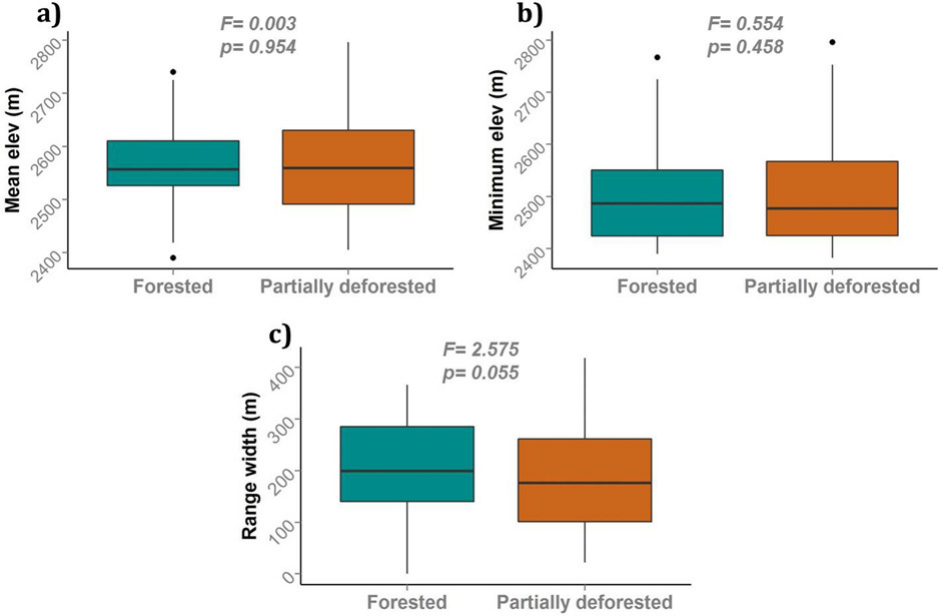
Comparison of elevational range variables for forested and partially deforested transects (p values shown are for one-factor ANOVAs). a) Abundance-weighted mean elevation. b) Minimum elevation. c) Elevational range width. Dots represent outliers.

We predicted that species’ mean elevational ranges and minimum elevations would be higher and elevational ranges narrower in partially deforested transects. A comparison of 134 species shared by forested and partially deforested transects showed no statistically significant differences between the abundance-weighted mean and minimum elevations (Fig 2A and 2B). Fig 2C shows that species in forested transects had mean range width 208±77 m, while those in partially deforested transects had marginally narrower elevational ranges with average 186±83 m (F = 2.575, p = 0.055).An additional 26 species, not included in the analyses, had at least two observations in forested transects but only one in partially deforested transects (13 species), or at least two in partially deforested transects but only one observation in forested transects (13 species).

**Fig 3:**
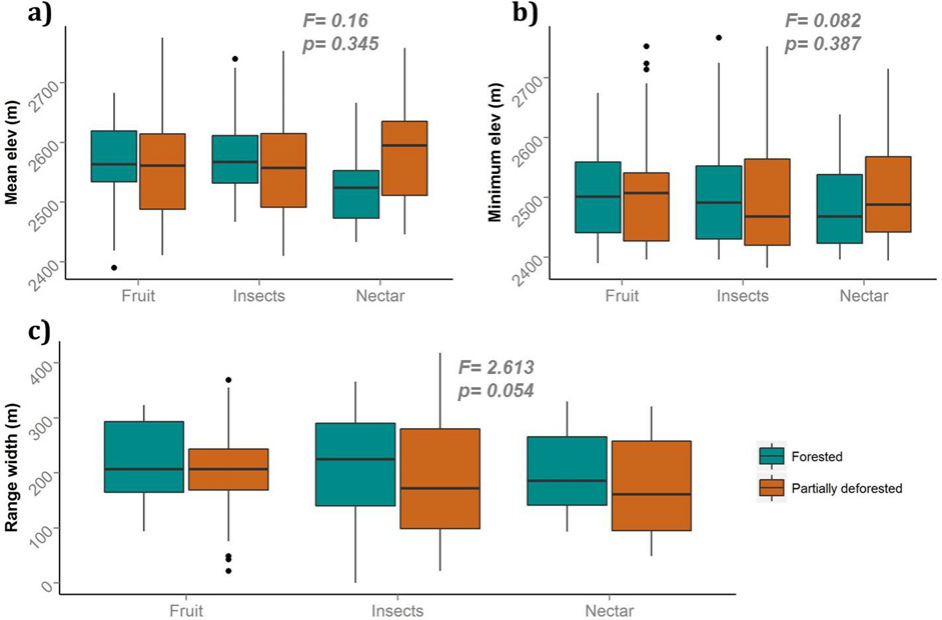
Comparison of elevational range variables per trophic guild for forested and partially deforested transects (p values shown are for one-tailed two-factor ANOVAs). a) Abundance-weighted mean elevation. b) Minimum elevation. c) Elevational range width. Dots represent outliers.

We tested if species of distinct trophic guilds or habitat preference categories showed significant differences in their elevational range variables. Fig 3 shows the elevational range variables for different trophic guilds for forested and partially deforested transects. Results from two-factor ANOVAs showed no statistical significance of trophic guild in the abundance-weighted mean and minimum elevations of the two types of transects (Fig 3A and 3B). It is important to note, however, that nectar-eating birds had considerably higher abundance-weighted mean elevations in partially deforested transects than other guilds. Elevational range width, in contrast, was marginally significantly different between forested and partially deforested transects, but the differences were explained mostly by the type of transect (F = 2.613, p = 0.054), and not the trophic guild (F = 0.708, p = 0.494) (S2 Table). Fruit-eating birds showed no discernible trend, while insect and nectar-eating birds had considerably wider ranges in forested transects. These results confirm those in Fig 2C, in that forested transects ranges are wider.

**Fig 4:**
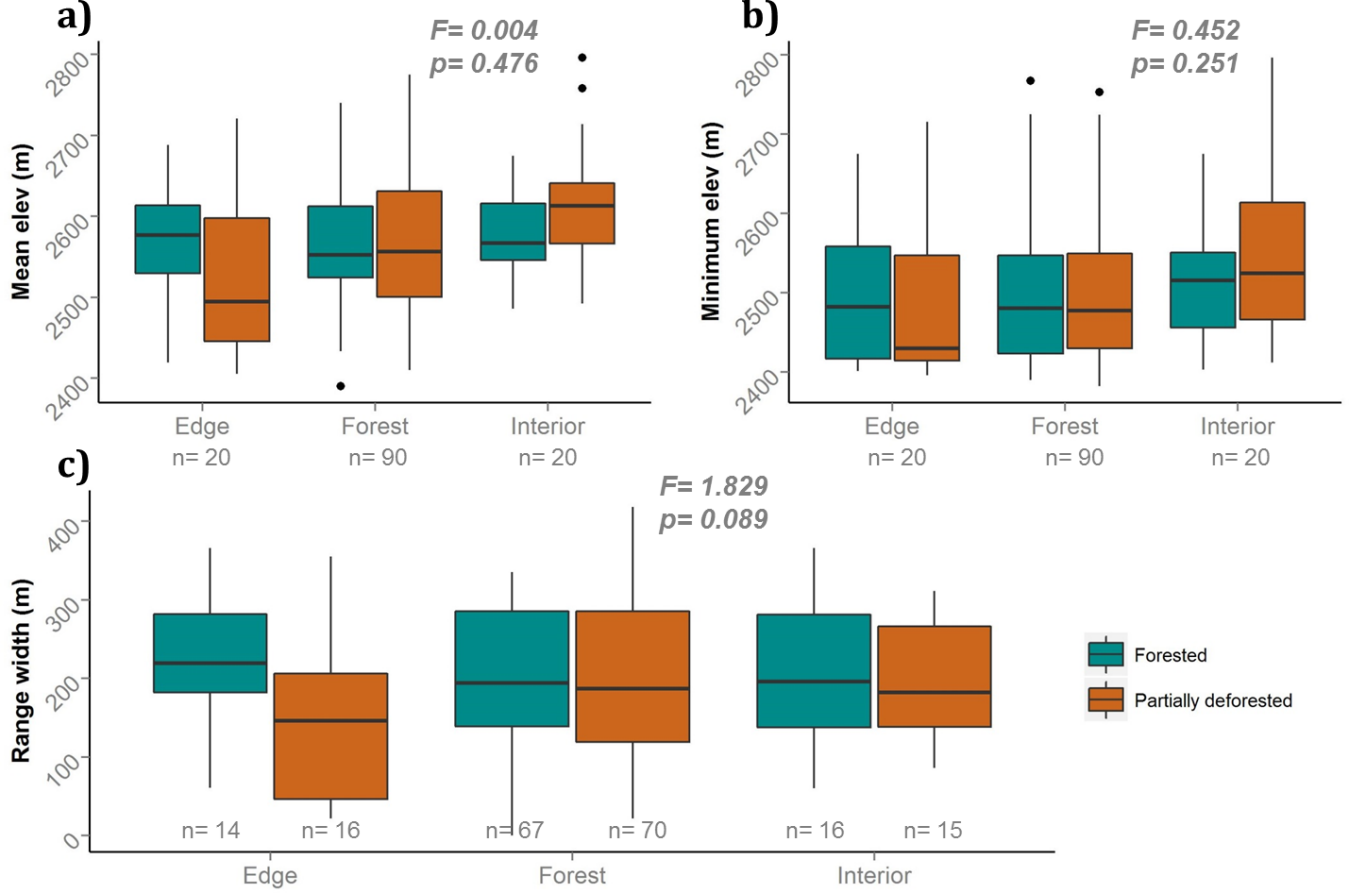
Comparison of elevational range variables per habitat preference category for forested and partially deforested transects (p values shown are for one-tailed two-factor ANOVAs). a) Abundance-weighted mean elevation. b) Minimum elevation. c) Elevational range width. Dots represent outliers.

Habitat preference categories were more important than trophic guilds when we analysed the differences between transects. Abundance-weighted mean was significantly different between habitat preference categories (F = 4.654, p = 0.01), regardless of transect type (F = 0.004, p = 0.476) (S3 Table). Within the habitat preference categories, a post-hoc Tukey test showed the strongest differences in abundance-weighted mean elevation were between interior-edge (p = 0.013) and interior-forest species (p = 0.024) (S4 Table). Interior birds had means at generally higher elevations in both transects, but highest in partially deforested transects. We found edge species at lower mean elevations in partially deforested transects (Fig 4A, S4 Table). Minimum elevation only showed a trend for edge species, which were at lower elevations in partially deforested transects. Elevational range width was marginally significantly different between transect type (F = 1.829, p = 0.089) but not between habitat preference categories (F = 0.295, p = 0.745) (Fig 4C, S5 Table).

## Discussion

We had expected abundance-weighted mean, and minimum elevations to be significantly higher in partially deforested transects in response to deforestation below 2400 m. When analysing species and trophic guilds, we found no significant difference in these elevational variables between transects. Habitat preference categories, in contrast, presented significant differences in abundance-weighted mean elevation, but habitat categories, not deforestation, explained these.

Range width, however, showed marginally significant differences in all analyses: per species, trophic guild, and habitat categories. These differences, though slight, are nonetheless consistent: overall range widths were larger in the forest transects (Fig 2C). They were larger for insect- and nectar-feeding species, essentially the same for fruit-eating species (Fig 3C), and also larger for edge, forest and interior species (Fig 4C).

First, we discuss why mean and minimum elevations were similar in forested and partially deforested transects. Two factors might explain this pattern:

1. Bird distributions are not only affected by forest cover locally, but also by the landscape configuration [53] and matrix [54]. Areas adjacent to our partially deforested transects had significant forest cover, so species probably moved to available nearby forests at similar elevation, instead of upslope. The existence of refuges can dampen the effects of deforestation since species that are affected by a disturbance can persist in the refugia, and then colonize larger areas that arise or exist nearby [55]. If our study was replicated in an area with higher rates of deforestation, we might see different results or even results in accordance to our predictions.
2. Different abiotic or biotic factors that we did not account for, at both local and global scales, could also affect the mean and minimum elevations of montane birds. These species might be tracking microclimates, may be displaced by competitors [56, 57], or respond to global scale climate change [58]. Range shifts due to habitat change are local and difficult to tease apart from other factors affecting species’ distributions. The amount of deforestation we used to classify our transect types (200m) might not be enough to separate impacts of deforestation on elevational ranges from those of the above-mentioned factors that also play a role.

### Range width

Now we discuss the impacts of deforestation on elevational range widths, in accordance to our predictions. Montane birds, regardless of species, trophic guild, and habitat preference had narrower elevational ranges when faced with deforestation below 2400 m (Figs 2C, 3C and 4C). Although the effects were marginally significant (p<0.1), they are consistent in their direction. We discuss this trend because it could have important conservation implications for these species.

Scientists have documented geographical and elevational range contractions of species in response to climate change [59, 60], and predicted alarming consequences [20, 58, 61]. Climate change could exacerbate the slight range contractions we found in montane birds. As a result of a large-scale model, [25] found an estimated 400 of 8750 species analysed should lose >50% of their current range due to land cover changes resulting from climate change.

Montane birds in our study site likely migrate altitudinally as studies on quetzals [62], bellbirds [63], and manakins [64] have documented in other tropical mountains. Altitudinal migrants in our forested transects had larger suitable areas for altitudinal movements, while partially deforested transects only offered a portion of that elevational span covered by forest. Looking at the records of our banded birds, we found evidence of 100–400 m altitudinal movement of birds along forested transects, which were scarcer in partially deforested transects. Range contractions resulting from deforestation could harm these, still unknown, migration patterns [65].

### Trophic guilds

Nectar-eating birds had higher mean and minimum elevations, and slightly narrower range in partially deforested transects (Fig 3A, 3B and 3C). We expected this response because birds that depend on highly variable and seasonal resources often need to move along larger areas and are more susceptible to fragmentation [66]. In this case, we suspect hummingbirds moved upslope in partially deforested transects in search of food. Although hummingbirds have also been described as less sensitive to fragmentation and crossing of forest gaps [13], their movements through landscapes are affected by forest availability, as a study of the Green Hermit (*Pheathornis guy*) showed [67].

We found insect-eating birds had considerably lower minimum elevations and narrower ranges in partially deforested transects (Fig 3A and 3C). These bird species preferred the edge of the forest in our study, probably due to higher availability of insects. Restrepo and Gómez (26) also found more insectivores near forest edges in wet months in a similar montane forest. While another study at similar elevations in Ecuador showed insectivores had highest densities in second growth and agricultural land, compared to primary forest [68]. This trend for montane insectivores contrasts with results for Amazonian forests that show loss of understory insectivores after fragmentation [69] but the life histories of these Amazonian birds are different from most Andean insectivores.

Fruit-eating species and, in general, differences between trophic guilds were not significantly different between transect types in our study (Fig 3). A study in the Atlantic Forest of Brazil found trophic guild to be a good predictor of vulnerability [70], and research from the Colombian Andes found large frugivores to be most vulnerable [65]. This similarity might be because the forests we studied offer a wide array of feeding resources. The 200m of deforestation might not be a significant loss of food resources for the species found in this forest, and they could also complement their diet by visiting the surrounding mostly forested matrix.

### Habitat categories

Our analyses of habitat preference categories showed edge specialists at lower elevations, and with narrower ranges, while interior birds were at higher elevations in partially deforested transects (Fig 4A, 4B and 4C). Birds that prefer forest interior are very vulnerable to fragmentation and habitat loss [68, 71]. As we predicted, in partially deforested transects the species in our interior habitat preference category were likely forced upslope in search for suitable habitat and away from harmful edge effects [72]. The similarity between mean and minimum elevations on forested and partially deforested transects might be a result of the large amounts of forest in this region. Studies in fragmented landscapes in both Andean forests [65], and Amazonia [27] have shown marked differences in species found in forest edges and interior. However, the geometry of a fragment and its edges are different from those of elevational transects where the edge is only found at one end of the forest. Because the majority of the species we classified in a habitat preference category were forest generalists, these species are probably driving the statistical tests and making it hard to see signals for edge and interior species.

### Recommendations

This study should be replicated in the future in areas with higher rates of deforestation, and comparing transects with a larger difference between forest availability (more than 200m), which may lead to different results. Ideally, an experiment could be conducted where the same transects are compared before and after lowland deforestation to document species loss over time and extinction debt. We recommend that researchers in the future control for other variables that could affect the results of this experiment such as food availability, plant composition and structure, microclimate, predator abundance, presence of invasive species, and other factors identified as important. This was the main limitation of our study. In addition, it was hard to tease apart the impact of lowland deforestation when the rest of the matrix was mostly forested, perhaps the impact would have a stronger signal in an area with a non-forested matrix.

### Conservation implications

Limited elevational range increases extinction risk [21, 73]. Species with narrow elevational ranges are more sensitive to landcover change and will be severely affected by climate change [36]. The conservation of forest along elevational ranges is essential for present and future species conservation. Protected areas would ideally provide continuous altitudinal corridors to allow upslope range shifts [22–24, 34, 74]. In addition, evaluation of species’ threat status should include elevational range width as a predictor for extinction, in addition to geographic range, as was also suggested by [21]. Studies that incorporate elevational ranges into the assessment of threat have found that species’ ranges are smaller than previously thought, and thus species are more threatened than their current categories suggest [17, 75].

## Conclusions

We compared elevational ranges of montane birds in altitudinal gradients with different amounts of forest between 2200 and 2800 m. Forested transects had forest from 2200 to 2800m, while partially deforested transects only had forest between 2400 and 2800m. Abundance-weighted mean and minimum elevations were not significantly different between forested and partially deforested transects. Range width was marginally but consistently different, with wider ranges in forested transects. Species of different trophic guilds and habitat preference categories showed different trends. These results suggest a small, but harmful impact of deforestation on species’ ranges, but we encourage further research. Future studies should include the assessment of other factors that could influence elevational distribution of species, as well as temporal comparisons of transects, and the evaluation of this effect within different landscape matrices.

## Supporting Information

S1 Table Bird species recorded in Mesenia-Paramillo Reserve during April 2014-March 2015. IUCN = threat categories [15]. End = Colombian endemic [76]. Inc = incidental observations, not within point counts. PC = point counts. MN = mist-nets. Mean = abundance-weighted mean elevation (m). Min = minimum elevation (m). RW = elevational range width (m). Taxonomy follows del Hoyo, Elliott (50).
(XLSX)

S2 Table Results from one-tailed ANOVA test comparing elevational range width of different trophic guilds, in forested and partially deforested transects. (DOCX)

S3 Table Results from one-tailed ANOVA test comparing abundance-weighted mean elevations of different habitat preference categories, in forested and partially deforested transects. (DOCX)

S4 Table Results from TukeyHSD test showing differences in abundance-weighted mean elevation between habitat preference categories, and forest and no-forest transects. (DOCX)

S5 Table Results from ANOVA test comparing elevational range widths of different habitat preference categories, in forest and no-forest transects. (DOCX)

S6 Table Abundance data for all species recorded at Mesenia-Paramillo Reserve during April 2014-March 2015 in alphabetical order. Numbers represent sum of 20 replicas for each point count (PC) in six elevational transects.
(XLSX)
