# Supplementary material for: Elevational Ranges of Montane Birds and Deforestation in the Western Andes of Colombia
Source: PLoS One. 2015 Dec 7;10(12):e0143311. doi: 10.1371/journal.pone.0143311 (PMC4671720; doi:10.1371/journal.pone.0143311)
Supplement: S4 Table — (DOCX) [file pone.0143311.s004.docx]

**S4 Table.**

| Habitat | diff | lwr | upr | p adj |  |
| --- | --- | --- | --- | --- | --- |
| Forest-Edge | 13.9 | -18.2 | 46.1 | 0.562 |  |
| Interior-Edge | 49.9 | 8.8 | 91.0 | 0.013 | * |
| Interior-Forest | 36.0 | 3.8 | 68.1 | 0.024 | * |
| Deforested-Forested | 0.6 | -18.6 | 19.7 | 0.477 |  |
|  | * p<0.05 | ** p<0.01 | *** p<0.001 | |  |
