# Supplementary material for: Elevational Ranges of Montane Birds and Deforestation in the Western Andes of Colombia
Source: PLoS One. 2015 Dec 7;10(12):e0143311. doi: 10.1371/journal.pone.0143311 (PMC4671720; doi:10.1371/journal.pone.0143311)
Supplement: S5 Table — (DOCX) [file pone.0143311.s005.docx]

**S5 Table.**

|  | Df | Sum Sq | Mean Sq | F value | Pr(>F) |  |
| --- | --- | --- | --- | --- | --- | --- |
| Habitat | 2 | 5345 | 2673 | 0.295 | 0.745 |  |
| Forest | 1 | 16551 | 16551 | 1.829 | 0.089 | . |
| Residuals | 194 | 1755846 | 9051 |  |  |  |
| *** p<0.001 | ** p<0.01 | * p<0.05 | . p<0.1 |  |  |  |
